# The learning curve of laparoscopic percutaneous extraperitoneal closure (LPEC) for inguinal hernia: protocoled training in a single center for six pediatric surgical trainees

**DOI:** 10.1186/s12893-019-0470-3

**Published:** 2019-01-14

**Authors:** Soichi Shibuya, Naho Fujiwara, Takanori Ochi, Momoko Wada, Toshiaki Takahashi, Kyeong Deok Lee, Eiji Miyazaki

**Affiliations:** 1 Department of Pediatric Surgery, Seirei Hamamatsu General Hospital, Shizuoka, Japan; 2 Department of Pediatric General and Urogenital Surgery, Juntendo University Hospital, 3-1-3 Hongo, Bunkyo-ku, Tokyo, 113-8431 Japan

**Keywords:** Pediatric surgery, Inguinal hernia, Learning curve, Laparoscopic surgery, Operative time, Surgical training

## Abstract

**Background:**

Laparoscopic percutaneous extraperitoneal closure (LPEC) has become a common procedure for repairing inguinal hernia. As a laparoscopic approach, pediatric surgical trainees require more training to learn LPEC than a traditional open approach. This study aimed to clarify the experience needed to acquire the skill to perform LPEC adequately.

**Methods:**

This descriptive single-center study used clinical data from patients who underwent LPEC between May 2009 and May 2016. The mean operative time for ten consecutive unilateral repairs was used as an index of proficiency with the procedure. The number of repairs performed before the mean operative time became less than 20 min was evaluated for each trainee.

**Results:**

During the study period, six pediatric surgical trainees participated in the training independently. The number of the patients was 987. The total number of repairs was 1436, including 538 unilateral repairs and 449 concurrent bilateral repairs. Overall, the mean operative time was 21.8 ± 8.1 min for unilateral repair and 31.4 ± 9.7 min for concurrent bilateral repairs. The mean number of repairs performed before the acquisition of skill for dexterous LPEC was 125.1 ± 29.5.

**Conclusions:**

Although there were individual differences, all trainees acquired the skill to perform LPEC adequately within one year. With appropriate guidance, LPEC can become a standard technique for pediatric surgical trainees, along with traditional open surgery. These results provide valuable information for planning LPEC training.

## Background

Laparoscopic percutaneous extraperitoneal closure (LPEC) was proposed by Takehara et al. for the first time as a laparoscopic repair technique for inguinal hernia [1]. LPEC is a simple technique that includes ligation around the internal inguinal ring, using a unique 19 G needle (LAPA-HER-CLOSURE™, Hakko®, Japan) that can easily hold and release threads with a wire contained inside the needle. LPEC can avoid opening the inguinal canal and limit dissection around the testicular vessels and the vas deferens minimally.

With the improvement of the technique and development of laparoscopic equipment, LPEC has become a standard procedure, especially in Japan. In the authors’ institution, LPEC has been performed on over 1500 patients since introduction in 2009.

Although LPEC is a simple technique, laparoscopic procedure has the potential for severe complications such as vascular and visceral injury. Therefore, similar to other laparoscopic procedures, young surgeons must be well-trained before performing LPEC. Compared with a traditional open approach, LPEC is thought to require surgical trainees more training to learn. However, only a few reports are describing the actual learning curve of LPEC.

This study aimed to investigate the actual learning curve and determine how much experience is needed to acquire the skill to perform LPEC adequately.

## Methods

This descriptive single-center study used clinical data from the patients who underwent LPEC between May 2009 and May 2016. During the period, six pediatric surgery trainees (Surgeon A - F) received the clinical training independently. The average of past clinical experience of the pediatric surgical trainees before training was 5.6 years (range, 4 to 9 years). Surgeon A, Surgeon B, Surgeon C, and Surgeon D had no experience of performing LPEC before the training. Surgeon E and Surgeon F had a few experiences in other institutions, which were assessed to be inconsiderable. After participating in over 20 surgeries as an assistant, they started to perform LPEC under the guidance of a consultant surgeon. The consultant surgeon remained the same throughout the study period. Mean duration of the training of the trainees was 13.1 months (SD: ± 2.6, range: 10–18), and there was no overlapping of the training period.

The operative information, including a profile of the patients, laterality of the hernias, operative time, and complications, was retrospectively collected. We used the mean operative time of 10 consecutive unilateral repairs (MOT) as an index of proficiency of each surgeon. MOT was the mean operative time of 10 consecutive surgeries in which only unilateral repair was performed, excluding surgeries in which simultaneous other procedures (repair for umbilical hernia, orchidopexy) or bilateral repairs were performed. The learning curves were plotted based on MOT and the number of repairs performed by each surgeon. The number of repairs was counted as one for unilateral repairs and counted as two for bilateral repairs. The number of repairs performed by each surgeon before the MOT became less than 25 min and MOT became less than 20 min and the learning curve plateaued were evaluated and compared.

The patients’ demographics among each group were compared using Analysis of Variance (ANOVA) test. The distribution of continuous data was evaluated using the Student *t*-test.

### Operative procedure

All the LPEC procedure was performed under general anesthesia with tracheal intubation.

**Fig. 1:**
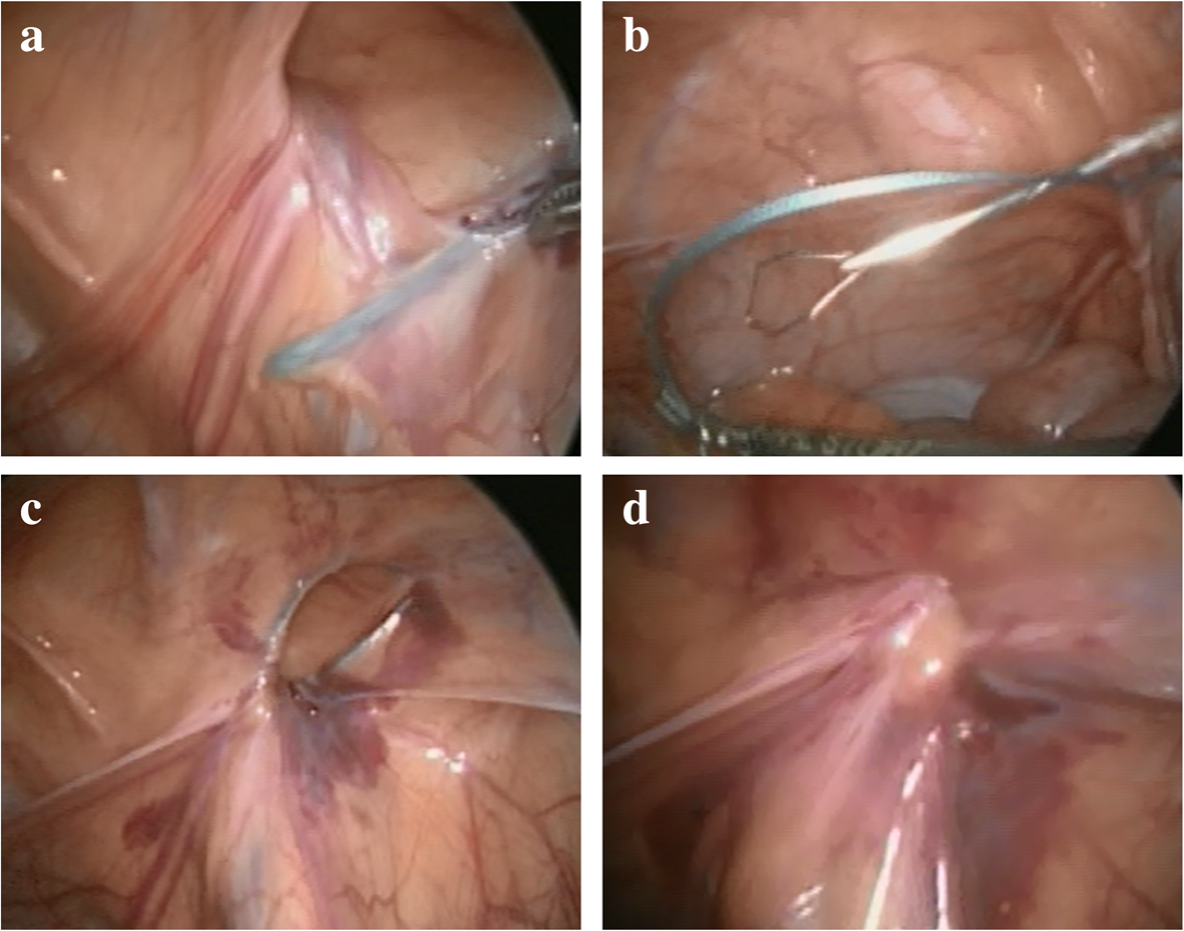
The operative procedure. **a**: LAPA-HER-CLOSURE® □ being advanced along the lower edge of the internal inguinal ring. **b**: The thread left in the peritoneal cavity were caught with the needle. **c**: Extracorporeal ligation. **d**: Completely closed hernia orifice

Firstly, 3 mm bladeless trocar was inserted through a small longitudinal incision made within the umbilicus. Pneumoperitoneum was maintained in 5 - 8 cm H2O with CO2 flow. After confirming no adhesion between the peritoneal wall and the intestine, another 3 mm trocar was inserted at the right flank region. The operative bed was tilted to the 15 degrees’ head down to clear the intestine from near the internal rings. The existence of an inguinal hernia or a patent process vaginalis (PPV) was carefully inspected on both sides. If there was patency of the internal ring, a special needle (LAPA-HER-CLOSURE™) was inserted to the retroperitoneal space on the upper lateral of the internal ring, following injection of local anesthesia and a slight skin incision (Fig. 1-a). The needle held two threads of non-absorbable sutures (2–0 Ethibond for ≧ one year, 3–0 Ethibond for < 1 year). Then, the needle was slid in the layer between the peritoneum and pre-peritoneal fascia semi-circumferentially below the internal ring. Once, the tip of the needle reached the contralateral side of the internal ring, the peritoneum was penetrated with the tip of the needle, and the pair of the thread was left in the peritoneal cavity. Then, the needle was pulled back to the point of insertion and slid semi-circumferentially above the internal ring to the penetrated hole. The pair of thread left in the peritoneal cavity were caught with the needle and pulled out through the retroperitoneal space (Fig. 1-b). The pair of threads were ligated one by one, and the inguinal ring was closed entirely (Fig. 1-c). Finally, the surgeon carefully confirmed that there is no slackening of the ligature, no skipping of the peritoneum, and no injury of the viscera (Fig. 1-d).

In female patients, the round ligament was ligated together with the peritoneum unless the ovary and the fallopian tube were not involved. In male patients, the testicular vessels and the vas deferens were passed about with particular attention.

The patients older than three months were discharged on the same day, while the patients of three months and younger were discharged after overnight observation. All patients were observed at the outpatient a week after surgery. They were told to revisit the hospital when they find any abnormality.

## Results

During the study period, 1531 patients underwent LPEC. Among them, 987 patients who underwent LPEC performed by the pediatric surgical trainees were included in the study. Regarding remaining 544 cases, the consultant surgeon performed LPEC on 520 patients and other surgeons performed LPEC on 24 cases. The mean age at surgery was 4.4 (± 3.0) years old. The sex distribution was 485 males (49.1%) and 502 females (50.9%).

Bilateral repair was performed in 449 patients (45.5%) Among the unilateral cases, right side repair was performed in 314 patients (58.4% and left side repair was performed in 224 patients (41.6% The mean operative time of bilateral repair and unilateral repair was 33.7 min (± 13.3) and 24.3 min (± 12.0), respectively. Regarding the repairs performed by the consultant surgeon, the mean operative time of bilateral repair and unilateral repair was 30.5 min (± 10.5) and 19.5 min (± 5.6), respectively. There was no significant difference between the mean operative time of bilateral repair performed by the trainees and the consultant (*p* = 0.057), while the mean operative time of unilateral repair performed by the consultant was significantly shorter than that by the trainees (*p* <  0.001).

There were no significant complications such as arterial bleeding and injury of abdominal viscera. There was no conversion to the open procedure. Two patients (0.2%) developed recurrence three years after the initial surgery, and the consultant surgeon performed the re-operation. Elevation of the testis was observed in one patient, but it did not require additional surgery.

Surgeon A - F performed the mean of 239.3 repairs (SD: ± 59.4, range: 179–351). There was no significant difference in the distribution of sex and laterality of the repairs among the groups (Table 1).

**Table 1:** Clinical and demographic data

Surgeons	A	B	C	D	E	F	*p*-value
	*n* = 179	*n* = 207	*n* = 269	*n* = 351	*n* = 184	*n* = 246	
Gender							
Male	68	92	143	162	70	138	N.S.
(%)	(38.0)	(44.4)	(53.2)	(46.2)	(38.0)	(56.1)	
Female	111	115	126	189	114	108	N.S.
(%)	(62.0)	(55.6)	(46.8)	(53.8)	(62.0)	(43.9)	
Age at the surgery (years)	4.3	4.4	4.0	4.6	5.1	4.4	N.S.
	(± 2.8)	(± 2.6)	(± 3.1)	(± 2.9)	(± 2.8)	(± 3.4)	
Height at the surgery (cm)	98.9	99.6	94.9	101.7	105.5	98.2	N.S.
	(± 20.9)	(± 20.7)	(± 25.9)	(± 20.9)	(± 19.3)	(± 26.3)	
Weight at the surgery (kg)	16.1	16.0	15.1	17.0	17.9	16.6	N.S.
	(± 7.0)	(± 7.6)	(± 8.3)	(± 8.0)	(± 7.2)	(± 9.3)	
Laterality							
Right-sided	85	117	143	191	98	129	N.S.
(%)	(47.5)	(56.5)	(53.2)	(54.4)	(53.3)	(52.4)	
Left-sided	94	90	126	160	86	117	N.S.
(%)	(52.5)	(43.5)	(46.8)	(45.6)	(46.7)	(47.6)	

Tables 2 and 3 shows the mean operative time of unilateral repair and bilateral repairs. Although each trainee showed some preference for the side of repair, there was no significant difference in operative time between right side repair and left side repair. The mean operative time in female patients was shorter than male patients.

**Table 2:** Operative times of unilateral repair

Surgeons	A	B	C	D	E	F
	*n* = 55	*n* = 89	*n* = 107	*n* = 145	*n* = 62	*n* = 80
Sex						
Male (min)	25.1	26.4	20.7	23.7	23.6	21.1
	(± 8.9)	(± 7.3)	(± 7.6)	(± 8.7)	(± 8.4)	(± 4.9)
Female (min)	23.8	24.4	18.2	20.1	21.0	18.2
	(± 9.1)	(± 8.3)	(± 4.2)	(± 5.1)	(± 6.8)	(± 5.0)
*p*-value	*N.S.*	*N.S.*	< 0.05	< 0.01	*N.S.*	< 0.01
Laterality						
Right-sided (min)	25.8	25.2	18.9	22.8	21.5	19.6
	(± 10.9)	(± 7.9)	(± 6.2)	(± 8.5)	(± 7.3)	(± 5.0)
Left-sided (min)	23.4	26.0	20.8	20.9	23.1	20.7
	(± 7.3)	(± 7.7)	(± 6.9)	(± 5.3)	(± 8.1)	(± 5.2)
*p*-value	*N.S.*	*N.S.*	*N.S.*	*N.S.*	*N.S.*	*N.S.*

**Table 3:** Operative times of bilateral repair

Surgeons	A	B	C	D	E	F
	*n* = 62	*n* = 59	*n* = 81	*n* = 103	*n* = 61	*n* = 83
Sex						
Male (min)	31.4	43.8	29.6	32.4	37.3	33.6
	(± 9.6)	(± 11.3)	(± 6.9)	(± 6.2)	(± 8.3)	(± 10.0)
Female (min)	29.1	37.8	27.7	27.4	33.5	28.8
	(± 12.3)	(± 12.3)	(± 7.9)	(± 6.0)	(± 8.8)	(± 9.2)
*p*-value	*N.S.*	< 0.05	*N.S.*	< 0.01	*N.S.*	< 0.05

**Fig. 2:**
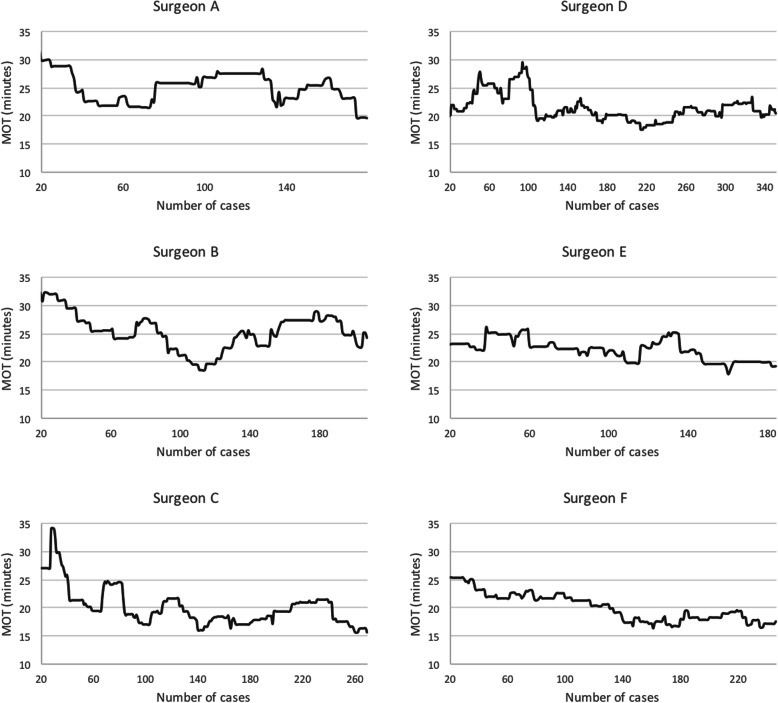
The learning curve of each surgeon. The numbers of repairs performed by Surgeon A - F before the MOT became less than 20 min and the learning curve plateaued were 174, 107, 84, 108, 147, and 131, respectively. The mean was 125.1 ± 29.5. MOT: Mean operative time of 10 consecutive unilateral repairs

Figure 2 shows the learning curve of each trainee. The mean of the number of repairs performed and duration of training before the MOT became less than 25 min was 48.2 (SD: ±27.7, range: 16–102), and 3.4 months (SD: ±1.5, range: 1.2–6.3), respectively. The mean of the number of repairs performed and duration of training before the MOT became less than 20 min and the learning curve plateaued was 125.1 (SD: ±29.5, range: 84–174), and 7.3 months (SD: ±1.7, range: 4.9–10.1), respectively.

## Discussion

Although laparoscopic inguinal hernia repairs in children have been proven to be safe and effective, controversy remains regarding whether it is adequate for pediatric surgical trainees to perform the procedure without enough experience for laparoscopic surgery [2]. There are several kinds of procedures for laparoscopic inguinal hernia repair including intracorporeal suture and extracorporeal approach [2–4]. While some of the procedures that require an intracorporeal suturing technique seem to be difficult for young trainees, LPEC is a simple procedure with less handling in the abdomen.

In this study, all trainees acquired the skill to perform LPEC adequately within ten months. During the training, the trainees improved their skills with a steady learning curve. That suggests LPEC can be adapted as a standard procedure for inguinal hernia repair without requiring special abilities. However, there was a variety of fluctuation in learning curves after the trainees acquired competency. That suggests some of the trainees became more careful to perform the procedure after experiencing a certain amount of cases. There was significant distance between the mean number of repairs required before MOT became 25 min (48.2 repairs) and that before MOT became 20 min (125.2 repairs). These results suggested the feature of learning curve of LPEC that relatively easy to get used to but hard to become proficient at. The learning curve of some surgeons didn’t plateau completely, suggesting surgeons may fall into pitfalls even after they get used to LPEC.

There were no significant complications, and the recurrence was very rare (0.2%), which was comparable to previously reported LPEC recurrence rates [4–6]. It means the safety of the surgery was ensured during the training. However, the supervision of the consultant surgeon is mandatory, as laparoscopic surgery contains a potential risk of severe complications if support is inadequate.

There were differences between the operative time for males and for females, which were significant in unilateral repair by Surgeon C, D, and F and bilateral repair by Surgeon B, D, and F. It is no wonder that repair in males is more difficult than in females, because differences in anatomy exist between males and females.

During the procedure, the surgeons manipulate the needle in the right hand and the forceps in the left hand; coordinated movement of both hands is most important for smooth operation. In the right-sided repair, the surgeon pushes the needle towards the abdominal cavity, while in the left-sided repair, the surgeon pulls the needle towards the abdominal cavity. Additionally, the angle of the forceps to grasp the peritoneum is different between right-sided repair and left-sided repair. Therefore, technical difficulty is different between right-sided repair and left-sided repair. Interestingly, though not significant, some trainees were faster in right-sided repair, and the other trainees were faster in left-sided repair. As all the surgeons including the consultant were right-handed, this difference might come from the habit of the surgeons in handling the needle.

According to the results, years of experience as a pediatric surgeon have little influence on the learning curve for LPEC. It means the LPEC technique, being relatively specific, can be acquired by any trainee. Therefore, it is of value for pediatric surgery trainees to start training for performing LPEC as soon as possible. To put it another way, even experienced surgeons require specific training before they become to be able to perform LPEC adequately.

It is the most substantial report describing the individual learning curve of LPEC by evaluating the operative time. Although operative time does not reflect the operative skill as it is, smoothly performing the procedure is essential for the safety of the surgery. Because taking time to achieve the procedure causes edema and bleeding in the retroperitoneal space and distinguishing the testicular vessels and the vas deferens becomes difficult.

Yoshizawa et al. also reported the learning curve of LPEC with the conclusion that residents require about 30 operations to perform LPEC safely, which was fewer times than our results [7]. It was because their standard point was 30 min, which was more lenient than our study. We arbitrary set the standard for 20 min because the mean operative time of unilateral repair of the consultant surgeon was about 20 min. We consider that our strict standard is more feasible because we must ensure the safety of the surgery even during the training.

There were some limitations in this study. As there was no significant difference in sex rate of the cases among each surgeon, we did not separate the operative time for male patients and that for female patients. To make it stricter, we should evaluate these separately. Additionally, we did not take into consideration the past experiences in other laparoscopic surgeries except for LPEC, which might have some influence on the learning curve.

## Conclusions

Although there were individual differences, all trainees acquired the skill to perform LPEC adequately within ten months. With appropriate guidance, LPEC can become a standard technique for pediatric surgical trainees, along with traditional open surgery. These results provide valuable information for planning LPEC training for pediatric surgical trainees.

## Abbreviations

LPEC: Laparoscopic percutaneous extraperitoneal closure
MOT: Mean operative time of 10 consecutive unilateral repairs
